# Dynamic and electrophoretic light scattering measurements on microtubules at low concentrations

**DOI:** 10.1371/journal.pone.0312430

**Published:** 2024-12-31

**Authors:** Annitta George, Ernesto Alva, Lorenzo Brancaleon, Marcelo Marucho

**Affiliations:** Department of Physics and Astronomy, The University of Texas at San Antonio, San Antonio, Texas, United States of America; Advanced Materials Technology Research Institute, National Research Centre, EGYPT

## Abstract

The accurate characterization of microtubules is essential for understanding their roles in various biological activities in eukaryotic cellular processes. *In vitro*, experimental data on these systems often need more details and information on sample preparation protocols and experimental techniques. This deficiency leads to unreproducible or contradictory outcomes. The use of diverse experimental methods and preparations yields different results of hydrodynamic and electro-mechanical properties, complicating the process of obtaining meaningful data and conclusive information. This article presents a robust and detailed protocol for performing dynamic light scattering (DLS) and electrophoretic light scattering (ELS) measurements on microtubules at low concentrations. This method ensures accurate and reproducible results on essential microtubule filament parameters such as the diffusion coefficient (*D*) and electrophoretic mobility (*μ*) from which other structures’ hydrodynamics, electrical, and stability properties can be elucidated.

## Introduction

Microtubules are highly charged polydisperse semiflexible polyelectrolytes formed by the polymerization of tubulin subunits [1–3]. These cytoskeletal filaments can dynamically rearrange and modify their conformations in response to changes in the surrounding environment, such as variations in electrolyte concentration and the type of crosslinker or binding proteins. This dynamic behavior is driven by their hydrodynamic, mechanical, and electrostatic properties [2,4–8]. The dynamic nature and conformational flexibility of cytoskeleton filaments are crucial for eukaryotic cells to fulfill specific biological functions in various cellular compartments. These functions vary depending on the cell type and its conditions [9,10]. Most experimental works on cytoskeleton filaments are based on optical treatments such as optical tweezers [11,12], rheology [13], electron microscopy [14–16], and many more [17], which alter the sample’s inherent characteristics. Laser light scattering studies comprise a versatile suite of non-invasive techniques that offer a distinctive advantage over previously mentioned experiments. They are conducted under hydrodynamic conditions, meaning that the system is immersed in liquid environments, particularly in a buffer system closely mimicking physiological conditions. They provide greater relevance and applicability to biological systems, allowing the investigation of the behavior of proteins in a more realistic and representative atmosphere. Modern dynamic light scattering (DLS), and electrophoretic light scattering (ELS) instruments use advanced technology and multi-functional software for characterizing particles (macromolecules and a wide range of nanometric materials) in aqueous solution [9,18–21].

DLS measures the intensity auto-correlation function of the laser light scattered by the particles in solution that form a random diffraction pattern and fluctuate in time as they move in Brownian diffusion. Analysis of the time dependence of the experimental data yields the particles’ diffusion coefficients, from which their hydrodynamic size and distribution can be calculated using hydrodynamic theories. Meanwhile, ELS measures the Doppler shift of the laser light scattered by charged particles in a solution as they migrate due to the application of an electric field. These measurements enable the determination of their electrophoretic mobility, from which the zeta potential and effective charge can be calculated using electrokinetic theories [22,23].

This article provides a unique methodology based on fundamental biostatistical tools [9,24] and a measurement protocol with an optimized configuration to perform DLS [25,26]and ELS [18,27] experiments on polydisperse microtubule filament samples at small volumes. This approach has been used to characterize their hydrodynamic and electrostatic properties at low concentrations. This study is based on our previous work [9,21], where we reported step-by-step polymerization and light scattering measurement protocols for actin filaments. To provide consistency, here we highlight the critical changes introduced for microtubules.

## Materials and methods

The polymerization protocol for preparing microtubule samples is provided in the S1 File (DOI: https://dx.doi.org/10.17504/protocols.io.dm6gpzrqjlzp/v1). The ‘Instrument startup’ process is shared for conducting both DLS and ELS experiments. In all our trials, we performed DLS experiments before proceeding with ELS experiments.

### Instrument start-up

- Power on the Zetasizer ULTRA instrument.
- Launch the ZS Xplorer software.

### Standard operating procedure (SOP) for DLS measurements

**Fig 1:**
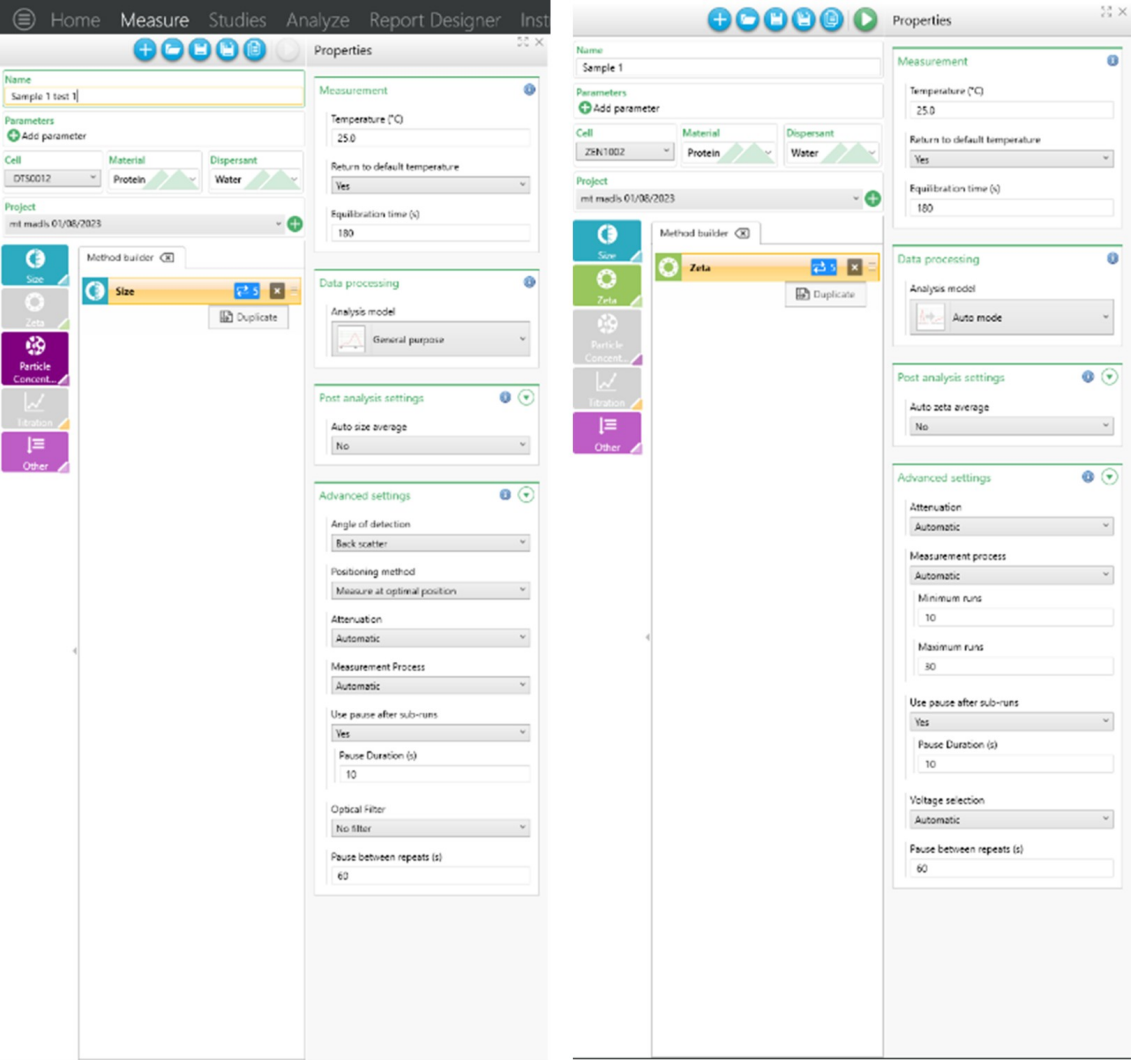
DLS and ELS settings. A) Screenshot for DLS settings in Malvern Zetasizer ultra as per SOP. B) Screenshot for ELS settings in Malvern Zetasizer ultra as per SOP.

All the specifications and measurement settings for the DLS experiments are tabulated in the following Table 1. See attached Fig 1A for reference.

**Table 1:** Standard operating procedure for DLS measurements.

Measurement Type	Selected Measurements
**Name**	Type a sample name for the measurement
**Cell**	DTS0012
**Material**	Protein
**Dispersant**	Water
**Project**	Select the project or create a new project
**Measurement type**	Size and 5 runs
**Temperature**	25°C
**Return to the default temperature**	YES
**Equilibration time (s)**	180
**Data Processing Analysis Model**	General Purpose
**Angle of Detection**	Backscatter
**Positioning Method**	optimal position
**Cell Position**	Automatically selected as 4.64
**Attenuation**	Automatic
**Measurement Process**	Automatic
**Use pause after sub runs**	YES
**Pause duration(s)**	10
**Optical Filter**	No Filter
**Pause between repeats (s)**	60

### Microtubule samples preparation for DLS experiments

**Fig 2:**
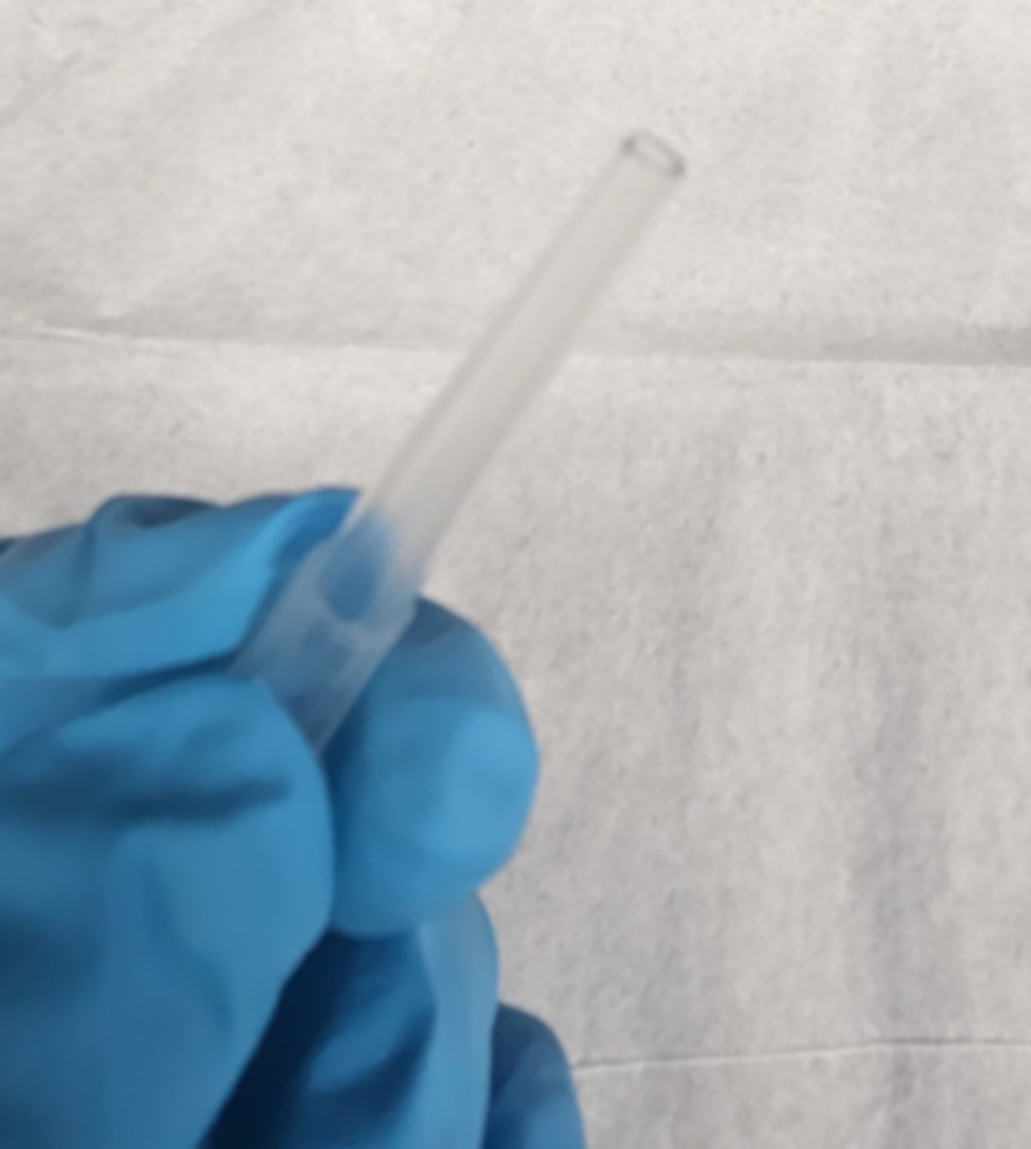
Cut tip. Illustration of a cut tip up to 3–5 mm diameter from the tip.

**Fig 3:**
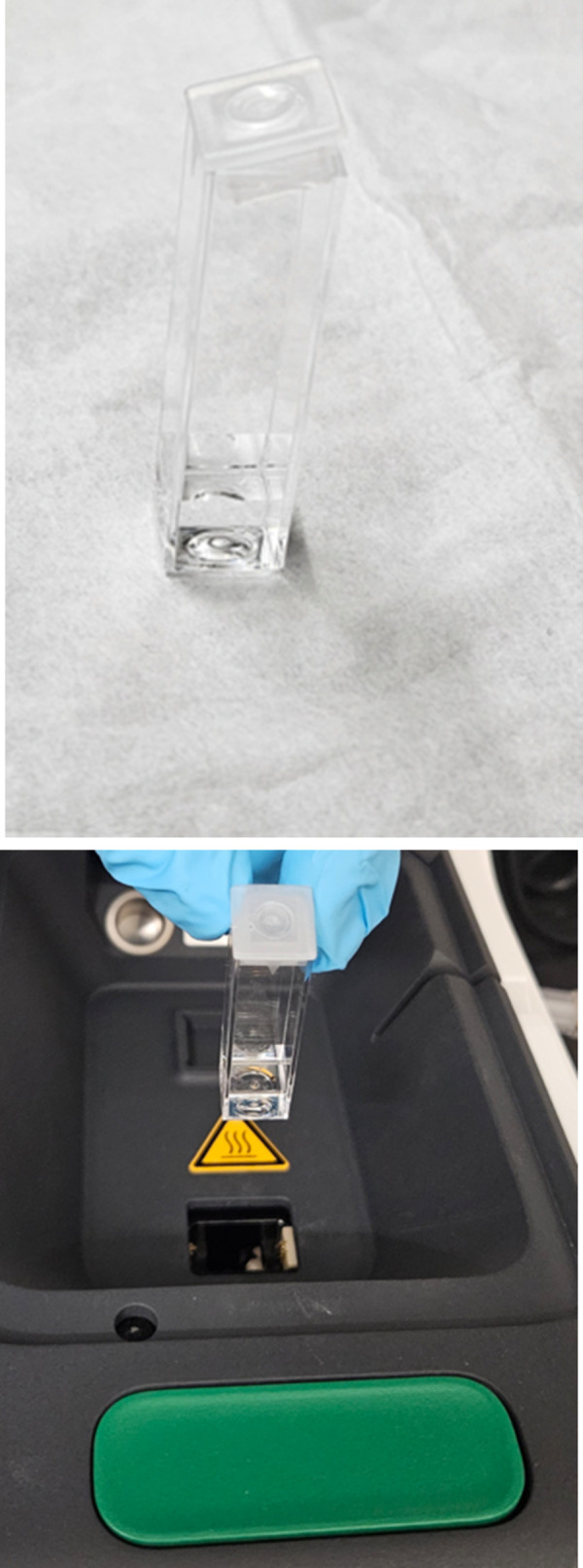
A) Partially capped cuvette. B) Place the sample into the Zetasizer instrument.

- Be ready with the 1 *ml* aliquots of polymerized microtubules, which we kept at room temperature.
- Meanwhile, clean and sterilize the scissors using water and ethanol. Remove/cut the tip of Optifit tips up to 3–5 *mm* diameter, as shown in Fig 2. This modification will significantly minimize filament breakage when transferring our protein solution into a cell cuvette for the experiments.
- Rinse the cuvette with ethanol and water three times and carefully dry it using air blowers to avoid leaving behind dust particles. The cell cuvette must be clean (free of dust and scratches, which could lead to inaccurate measurements).
- Set the Pipette+ (5–350 *μL*) titration mode at low speed (1–2/10).
- Dispense the protein solution into the cell cuvette by titrating the protein at a 45° angle mode to avoid forming air bubbles.
- Fully cap only one side of the cell, as shown in the Fig 3A. This step will minimize the occurrence of air bubbles during the measurements.
- Press the green button in front of the lid to open the cell holder. Place the cell cuvette into the cell holder. Ensure that the small triangle at the top of the cell faces the user. Close the cell area lid. See Fig 3B.
- To minimize the sedimentation, mix the protein solution 2–3 times using a cut tip after each round of data collection (after 5 consecutive runs) before proceeding to the next set of experiments.

### DLS measurements

**Fig 4:**
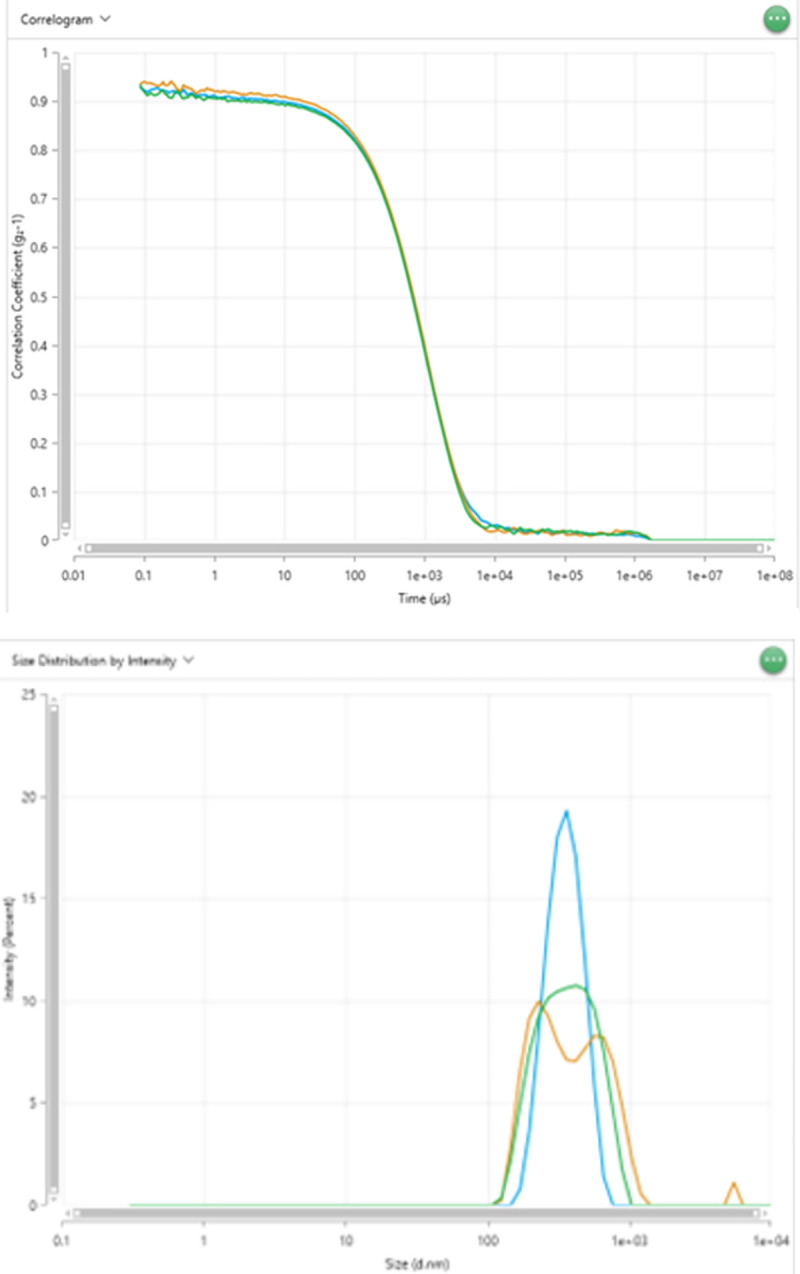
A) **Correlation coefficient plot**. An illustrative example of the correlation data function [g_2_ (t) -1 vs. time (*μs*)] plots obtained from size measurements. B) Size distribution based on intensity.

- Ensure the sample and measurement specifications are set according to the SOP. Then, click the Play button icon next to the property selection to initiate the measurements.
- Select multi-view to display the following three windows:
  1. “g_2_ (t)-1” versus decay time “t,” which updates continuously during the run (see Fig 4A)
  2. The intensity fluctuations.
  3. The intensity distribution (See Fig 4B).
- Navigate to the Analyze tab and locate the Project Name to access all data generated by the measurement. Extract data such as "correlograms," "Intensity vs. Size," and "Intensity vs. Volume."
- When the measurement is complete, the blue light will change to green.
- The Zetasizer ULTRA is ready for the next set of measurements.

### Standard operating procedure (SOP) for ELS measurements

All specifications and measurement settings for the ELS experiments are tabulated in Table 2. See attached Fig 1B for reference.

**Table 2:** Standard operating procedure for ELS measurements.

Measurement Type	Selected Measurements
**Name**	Type the sample name for the measurement
**Cell**	ZEN1002
**Material**	Protein
**Dispersant**	Water
**Project**	Select the project
**Measurement type**	Zeta and 5 runs
**Temperature**	25°C
**Return to the default temperature**	YES
**Equilibration time (s)**	180
**Data Processing Analysis Model**	Auto mode
**Attenuation**	Automatic
**Measurement Process**	Select Automatic with minimum runs set at 10 and maximum runs set at 30
**Use pause after sub runs**	YES
**Pause duration(s)**	10
**Voltage selection**	Automatic
**Pause between repeats (s)**	60

### Microtubule samples preparation for ELS experiments

**Fig 5:**
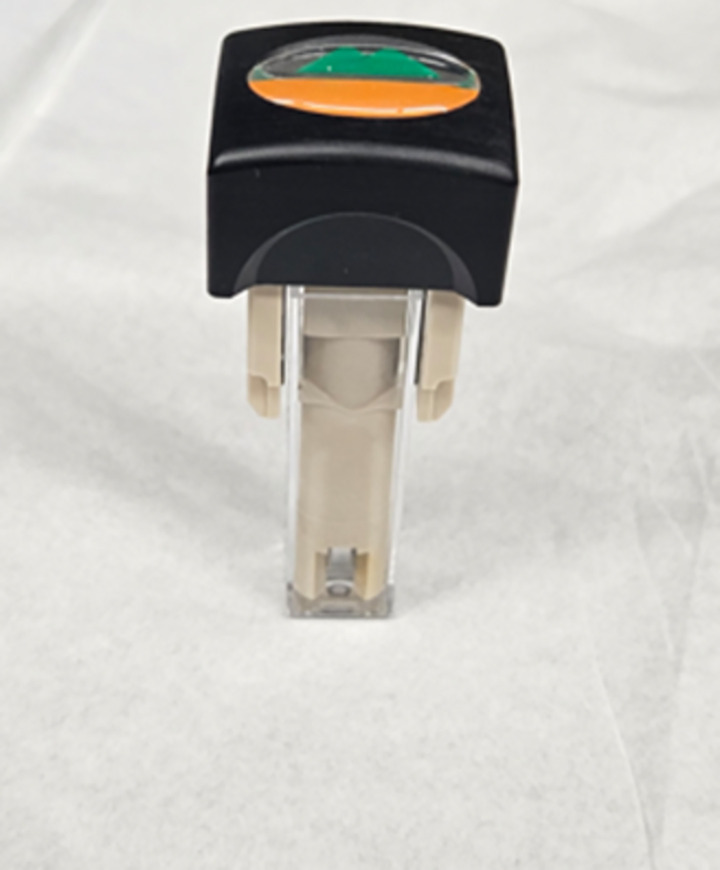
A sample with the dip cell to measure ELS.

- Before using the dip cell, its electrodes must be cleaned with ethanol and water. Alternatively, immerse the electrodes in a gentle ultrasound bath (30 *Watts*) for five to ten minutes before use. Electrodes must be cleaned after each measurement. Failing to do so will lead to cross-contamination of the samples.
- When the DLS measurement is completed, insert the dip cell/ZEN1002 into the cuvette, as shown in Fig 5. Ensure the sample does not overflow the cuvette when the ZEN1002 is fully inserted.
- Place the sample carefully in the cell holder.
- Close the cell area lid and proceed with the measurements.

### ELS measurements

**Fig 6:**
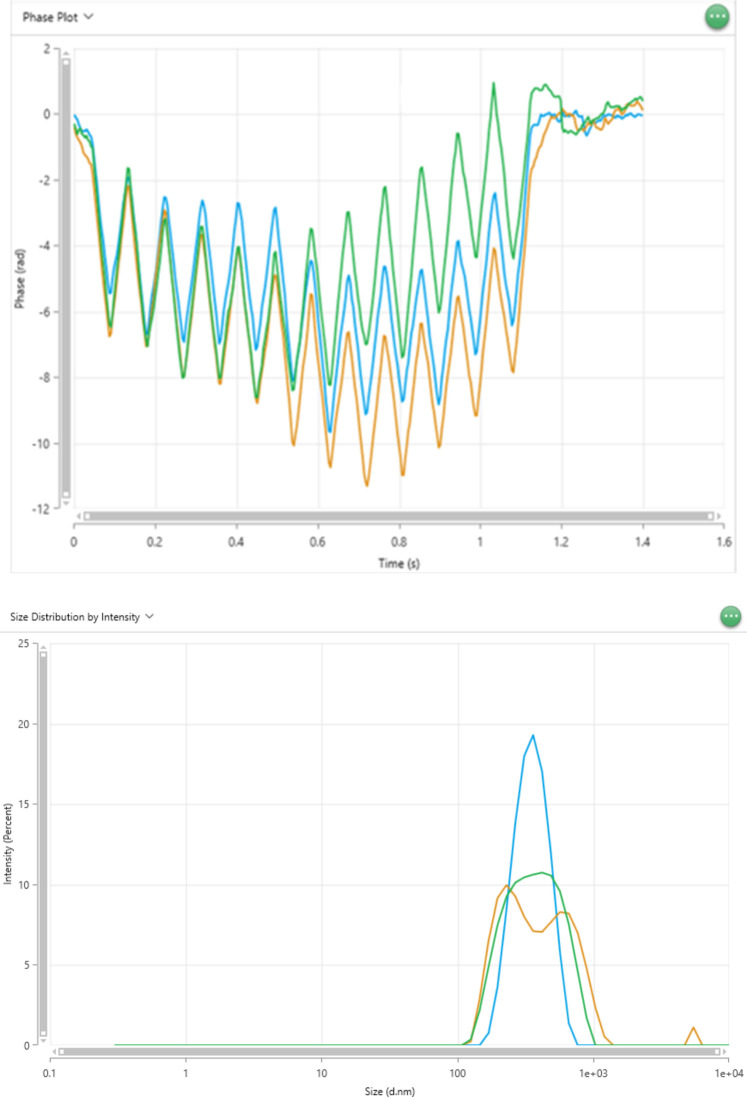
**A) Phase plot.** An illustrative example of phase plots obtained from electrophoretic measurements. **B) Frequency Shift plot.** An illustrative example of the frequency shift plot to reduce statistical errors in the electrophoretic mobility values.

- Ensure the sample and measurement specifications are set according to the SOP. Then, click the Play button icon next to the property selection to initiate the measurements.
- Choose multi-view to display the following three windows.
  1. “PALS: phase(rad) vs. t,” continuously updated during the run (see Fig 6A).
  2. The intensity fluctuations.
  3. The frequency shift (see Fig 6B).
- Navigate the Analyze tab and locate the Project Name to access all the data the measurement generates. Extract data such as “frequency shift” and “Phase plot.”
- When the measurement is complete, the blue light will turn to green.
- The instrument is ready for the next set of measurements.

## Results

### A. DLS analysis

**Fig 7:**
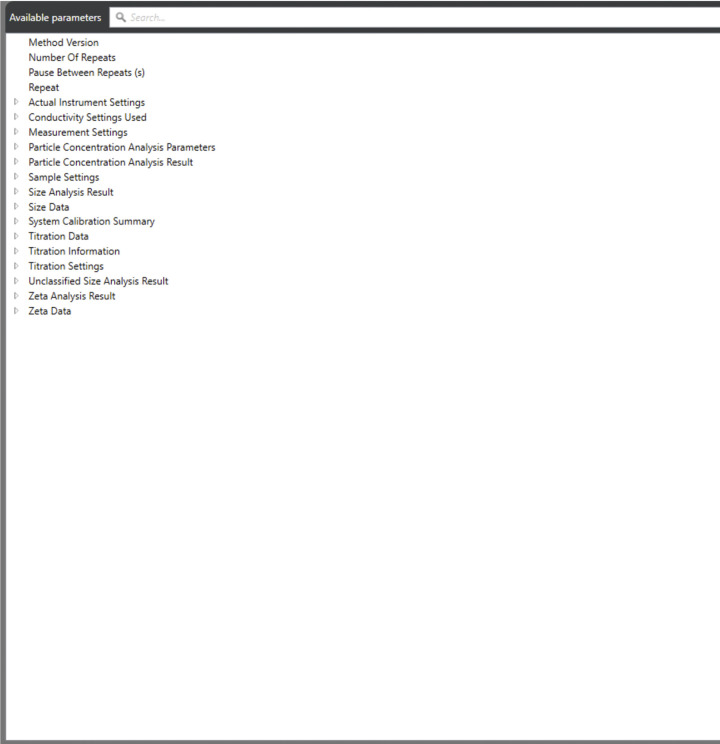
Table properties. Screenshot of the table properties available in the Xplorer software with all parameters.

**Fig 8:**
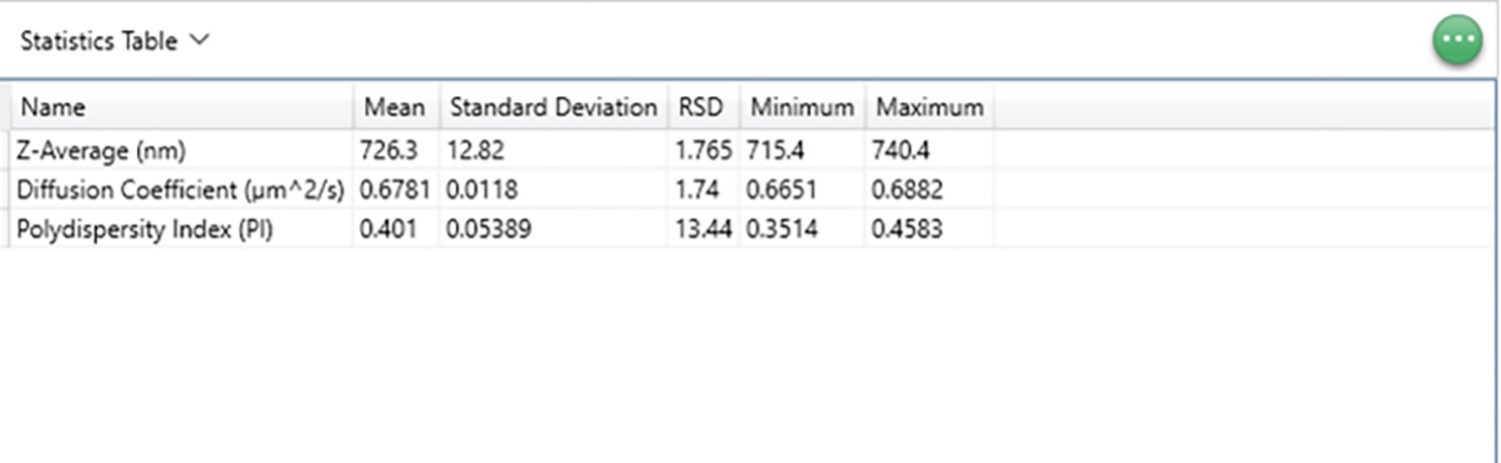
Statistics table for DLS measurements.

**Fig 9:**
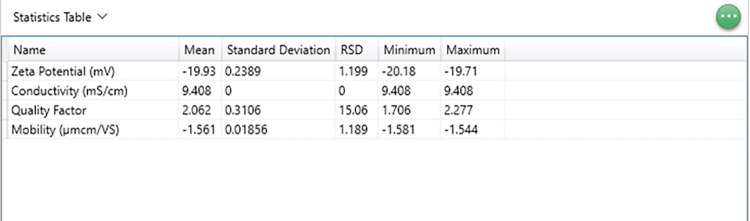
Statistics table for ELS measurements.

- Select the Size tab within the Analyze section, where all measurements are stored. In the data options, select the multiple parameters available for each run. Fig 7 provides a full display of available parameters and properties that can be selected based on the user’s requirements for DLS and ELS. Selected parameters recommended for DLS and ELS measurements are presented in parametric tables shown in Figs 8 and 9, respectively.
- Once the selected parameters are displayed (Fig 8), opt for the correlogram as one of the data plots for each run. The correlogram is a vital tool for assessing sample quality and measurement accuracy.
- The correlograms must meet the following experimental criteria to minimize errors and guarantee reproducibility.
  - The intercepts of the correlogram plot must be under 1.
  - The polydispersity index must be within the 0.4 < PDI < 0.8 range, indicating a good quality in the polydispersity samples.
  - The derived count rate must be higher than 100 *kcps*, (minimum value required for suitable measurements).
  - The correlogram plots must be a smooth, uniform single exponential decay (See Fig 4A). If the plots do not meet this criterion, the data may be erroneous due to dust, aggregates, and sedimentation. Such data should be disregarded to minimize error and enhance reproducibility.
- Size distribution by intensity indicates the relative contribution or weight of different particle sizes within a sample, as determined by the intensity of scattered light (see Fig 4B).
- Four independent samples with the same physiological conditions/buffer (pH:6.99) were used to run the DLS experiments at the backscattering angle. In each of these samples, the instrument measured five independent diffusion coefficients, and later, two of them were discarded to increase accuracy. The twelve experimental diffusion coefficient values were obtained and are summarized in Table 3. The S1 Dataset provides the unnormalized backscattering auto correlograms raw data (g_2_ (t) -1) necessary to replicate the diffusion coefficient values. We performed standard calculations to estimate the mean value and uncertainty of the diffusion coefficient and electrophoretic mobility [9,28].

**Table 3:** Experimental diffusion coefficient (D) values for buffer 1.

Buffer pH:6.99		
	x	x_avg_
**Diffusion coefficient *(μm^2^/s*)**	0.9404	0.9476 ± 0.1207 (3.48%)
	0.8058	
	1.069	
	1.013	
	1.118	
	0.9854	
	0.9259	
	0.7749	
	1.136	
	0.8357	
	0.8389	
	0.9278	

The second column lists the twelve diffusion coefficient values measured for each buffer. In contrast, the third column provides the mean value, the uncertainty (standard deviation of the mean value) [28], and the percentage error.

### B. ELS analysis

- Navigate to the Zeta tab within the Analyze section, where all the measurements are saved. In the data options, choose from multiple parameters available for each run. Fig 7 displays the parametric table with zeta potential, electrophoretic mobility, quality factor, and conductivity.
- Once the selected parameters are visible (Fig 9), opt for each run’s phase plot and frequency shift data plots. The fast field reversal (FFR) phase plot is a vital data function that determines the sample quality and measurement accuracy.
- The Phase plots must meet the following criteria to minimize errors and guarantee reproducibility.
  - The quality factor must be higher than one; if not, the data is discarded.
  - The phase FFR plot should have a sinusoidal behavior (see Fig 6A).
  - The frequency shift plots must have shallow traces of noise (See Fig 6B).
- We performed the ELS experiments using four independent samples with the same physiological conditions/buffer (pH:6.99). In each of these samples, the instrument measured five independent, consecutive electrophoretic mobilities, and later, two of them were discarded to increase accuracy. The twelve experimental electrophoretic mobility (*μ*) values are obtained and summarized in Table 4. The S2 Dataset provides the fast field reversal phase raw data necessary to replicate the electrophoretic mobility values.

**Table 4:** Electrophoretic mobility (μ) values obtained from ELS experiments.

Buffer pH: 6.99		
	x	x_avg_
**Mobility (*μm·cm/V·s*)**	-1.761	-1.599 ±0.1163 (3.36%)
	-1.797	
	-1.775	
	-1.513	
	-1.498	
	-1.484	
	-1.586	
	-1.598	
	-1.586	
	-1.508	
	-1.486	
	-1.593	

The second column lists the twelve measured electrophoretic mobility values for the buffer. In contrast, the third column provides the mean value, uncertainty (standard deviation of the mean value), and percentage error.

### C. Visualization of microtubules

**Fig 10:**
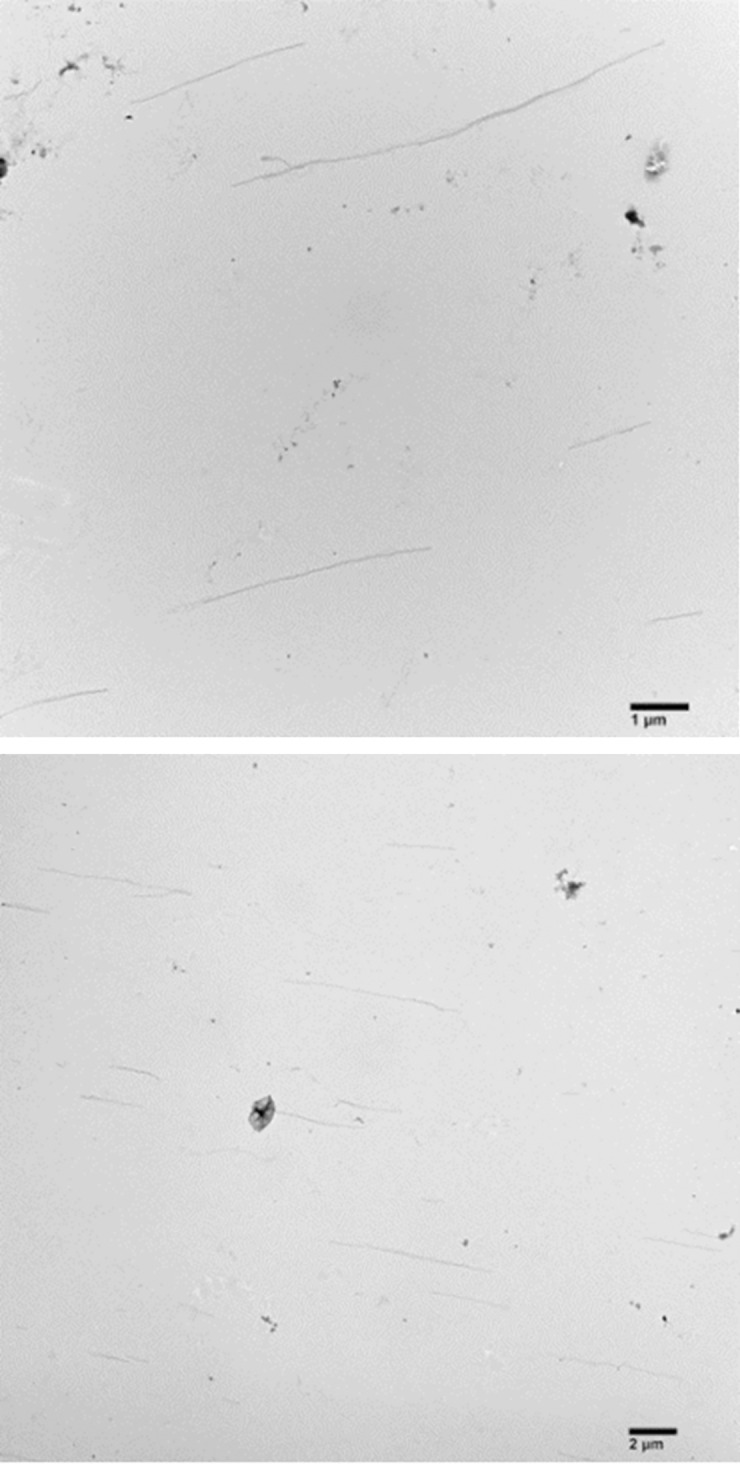
Micrographs of microtubule filaments. Illustrative images of microtubule filaments using the JEOL 1230 transmission electron microscope.

It is essential to perform micrographs to ensure the accuracy of the filaments and facilitate successful experiments. These microscopic images visually confirm the filament’s characteristics, allowing us to verify its identity and quality. An illustrative micrograph of microtubule filaments using the JEOL 1230 transmission electron microscopy is given in Fig 10. During the TEM sample preparations, the samples underwent a series of steps using 1% uranyl acetate on a formvar-coated grid. The samples were washed 2–3 times with distilled water. These micrographs are used only to validate that our experimental protocol for preparing the microtubule filaments is successful and reliable.

## Discussion

For experiments with the 1 *ml* sample solution, we utilized a Malvern ULTRA Zetasizer He-Ne 633 *nm* laser instrument, employing highly precise and non-invasive DLS and ELS techniques. The instrument measures the microtubule filament’s rate of Brownian motion (diffusion coefficient, *D*) and the electrophoretic mobility *μ*. We used the same sample and experimental conditions for DLS and ELS measurements to ensure accuracy and reproducibility. The experiments were configured, and the data was recorded and analyzed using the Zetasizer Xplorer software. This software offers an Adaptive Correlation algorithm that utilizes sample information to determine the optimal measurement duration, ensuring data consistency. This feature identifies and excludes enormous particles such as dust or aggregates, thereby retaining consistently present populations [9]. It provides high-sensitivity information on how filaments distribute themselves within the biological liquid, how the size and shape affect their hydrodynamic and mechanical properties, and how they interact with other charged particles, reflecting their electrical properties. In all our measurements, we utilized a 180-second equilibration time to stabilize the sample at a temperature of 25°C thermally. We selected the predefined “*protein”* material option with a refractive index of 1.450 and an absorption of 0.001 for microtubules. We chose the predefined *“water”* dispersant option with a refractive index of 1.33 and a viscosity of 0.8872 *mPa*.*s* for the buffer. It is essential to highlight that the refractive index and absorption of the material do not affect the Z-average, polydispersity, and intensity distribution results [21].

As shown in Fig 1, the purpose of using “runs” and “pause times” in the DLS and ELS settings serves purposes manifold. These include reducing sample heating, allowing the sample to recover to 25*°C* between successive measurements, minimizing sample degradation, and avoiding increasing mobility with sequential measurements. We manually chose minimum and maximum runs as 10 and 30, respectively. If a measurement requires more than 30 runs, it indicates that the sample is contaminated or aggregated, and the instrument cannot correlate the data. This approach enables real-time insights and more frequent data collection points. Additionally, we improved reproducibility and precision by incorporating a long pause (60 *seconds*) between consecutive measurements.

Furthermore, the attenuation factor was opted for ‘automatic’ allowing the laser power to be adjusted automatically so that the count rate from the sample is within acceptable limits. This setting offers 11 positions to control the beam intensity ranging from 100% to 0.0003%. The instrument exhibited an attenuation factor between 10 and 11 during DLS and ELS experiments. The automatic laser attenuation optimizes the count rate for the detector with high sensitivity and prevents damage from being overwhelmed during the measurement. The measurement duration was automatically determined from the detected count rate; a lower count rate resulted in a longer measurement duration and increased noise.

We placed 1 *ml* of microtubule filament solution for the DLS experiments in a 12 *mm* square Polystyrene cuvette (DTS0012). Correlation functions were measured at the back-scattering angle (173°), significantly reducing the effects of multiple scattering and dust by shortening the distance the incident beam travels through the sample. We used the ‘General Purpose’ analysis model using a CONTIN algorithm. It is the most suitable model for our case due to the unknown size distribution, which is the case in our previous work [9,21]. Also, we opted for no optical filter since our microtubule filaments are not fluorescent or not labeled with any dye, and no polarizers are required with our sample for further characterization.

We analyzed our DLS results following our experimental criteria in Results Section A(c). The analysis revealed that all correlation plots and intercepts were below one and within a low polydispersity index range of 0.3–0.5, indicating a good sample quality. Additionally, all our experimental size distribution measurements showed a derived count rate exceeding 100 *kcps*, the minimum value required for obtaining suitable measurements. Out of five consecutive runs, the three correlation data functions are selected for data analysis after two measurements (2/5) are disregarded according to the experimental criteria to increase accuracy. The selected correlogram or the correlation data (3/5) function plots are obtained from size measurements (see Fig 4A). The Z-average and polydispersity index (PDI) is derived from the correlation function of the scattered intensity based on the cumulant algorithm, wherein a single particle size is assumed, and a single exponential fit is applied to the autocorrelation function. The size distribution intensity plot (Fig 4B) elucidates the relative intensity or weight of the varied particle sizes present within our sample. The broad distribution of sizes confirms the polydispersity of our samples. An example of the parametric table, including the Z- average value, diffusion coefficient, and polydispersity index, along with average standard deviation and percent error for the backscattering angle, is shown in Fig 8. Also, we calculated the average of the three longitudinal diffusion coefficient values from five consecutive runs with the lowest standard deviation and best correlation function to minimize error and enhance reproducibility. The experimental average value of the diffusion coefficient of our four microtubule samples is listed in Table 3.

In the ELS experiments, we employed the Malvern Panalytical Universal dip cell kit (ZEN1002) and a 12 *mm* square Polystyrene cuvette (DTS0012) (Fig 5) to measure the electrophoretic mobility of microtubule filaments. The voltage selection and measurement process were set to automatic. Consequently, Zetasizer Xplorer software measures the sample’s electrical conductivity and adjusts the cell voltage to maintain a low current, approximately 5 *mS/cm*. This prevents the temperature rise near the electrodes, which causes bubble formation, sample degradation, and inaccurate data measurements. These automatic settings of the instrument minimize potential problems such as Joule heating, electrode degradation (blackening), and sample degradation (aggregation/denaturation).

The ‘auto mode’ setup in the software automatically selects either soft field reversal (SFR) or fast field reversal (FFR) based on the measured conductivity of the sample. The software will apply only fast field reversal if the sample conductivity exceeds 5 *mS/cm*. We concentrated on the FFR of the phase analysis light scattering (see Fig 6A) since the samples’ conductivity is higher (Fig 9). In FFR, the measured mobility is solely due to the particles’ electrophoresis and is unaffected by electro-osmosis associated with SFR. All our experimental electrophoretic mobility values were obtained with a quality factor above 1, exceeding the minimum value required to ensure good-quality data. Our frequency shift plots (Fig 6B) demonstrate additional evidence of data quality, which are noise-free and show consistent results. We repeated five consecutive runs for each microtubule filament sample. We chose three out of five to reduce statistical errors in electrophoretic mobility results, and the average value of the error is recorded in Table 4.

All the settings and analysis make our protocol easy to implement, time efficient, and capable of studying microtubules in their hydrodynamic state without extensive sample preparation. This protocol yielded reproducible and reliable results in the average values of diffusion coefficient and electrophoretic mobility in light scattering experiments, ensuring the preparation of stable, dispersed, and aggregate-free microtubule filament samples. The results for the average diffusion coefficient and electrophoretic mobility, tabulated in Tables 3 and 4, agree with findings in other research papers [29–31] thereby validating the reliability and accuracy of our approach. The slight discrepancies in values may arise from variations in *paclitaxel* concentration or buffer composition, temperature fluctuations, and disparities in the experimental setups.

Overall, in this article and the polymerization protocol, we addressed and meticulously explained many challenges in preparing microtubule samples. These include homogenizing microtubules without forming any clusters after centrifugation, stabilizing the microtubules by adding *paclitaxel* without causing precipitation, and preventing filament breakage while transferring samples to the cuvette.

## Supporting information

S1 File Protocol. (PDF)

S1 Dataset Unnormalized Back-scattering raw data required to reproduce the diffusion coefficient values provided in Table 3. (XLSX)

S2 Dataset Fast field reversal phase raw data required to reproduce the electrophoretic mobility values provided in Table 4. (XLSX)
