# Supplementary material for: Dynamic and electrophoretic light scattering measurements on microtubules at low concentrations
Source: PLoS One. 2024 Dec 31;19(12):e0312430. doi: 10.1371/journal.pone.0312430 (PMC11687780; doi:10.1371/journal.pone.0312430)
Supplement: S1 File — (PDF) [file pone.0312430.s001.pdf]

Sep 10, 2024

In vitro preparation of single filamentous microtubules optimized for dynamic and electrophoretic light scattering measurements.

DOI
dx.doi.org/10.17504/protocols.io.dm6gpzrqjlzp/v1

Annitta George[1], Ernesto Alva[1], Lorenzo Brancaleon[1], Marcelo Marucho[1]

[1]Department of Physics and Astronomy, The University of Texas at San Antonio, San Antonio, USA.

Annitta George
UTSA

OPEN ACCESS

DOI: dx.doi.org/10.17504/protocols.io.dm6gpzrqjlzp/v1

Protocol Citation: Annitta George, Ernesto Alva, Lorenzo Brancaleon, Marcelo Marucho 2024. In vitro preparation of single filamentous microtubules optimized for dynamic and electrophoretic light scattering measurements.. **protocols.io** https://dx.doi.org/10.17504/protocols.io.dm6gpzrqjlzp/v1

License: This is an open access protocol distributed under the terms of the **Creative Commons Attribution License**, which permits unrestricted use, distribution, and reproduction in any medium, provided the original author and source are credited

Protocol status: Working
We use this protocol and it's working

Created: April 03, 2024

Last Modified: September 10, 2024

Protocol Integer ID: 98285

Funders Acknowledgement:
NIH
Grant ID: 1SC1GM127187

Abstract

The lack of essential information on sample preparation and the need to characterize and understand microtubules (MTs) in various biological functionalities within the cell have led us to develop a high-quality polymerization protocol to prepare fully formed MTs in vitro, which is particularly designed for Dynamic light scattering (DLS) and Electrophoretic light scattering (ELS) experiments for further analysis. This protocol details the reconstitution of tubulin, GTP, and Taxol, the preparation of PEM, cushion buffer, and polymerization buffers, the step-by-step polymerization process, and the use of Taxol to stabilize the microtubule filaments without precipitation. This polymerization protocol successfully generated samples for light scattering experiments using buffers that replicate physiological conditions. This has provided consistency in preparing stable, diluted, aggregate-free, homogenous microtubule filament samples that could benefit many other scientific research groups currently working in the field. Additionally, it can easily be adapted to prepare samples using other buffers and biological fluids.

Graphical abstract:

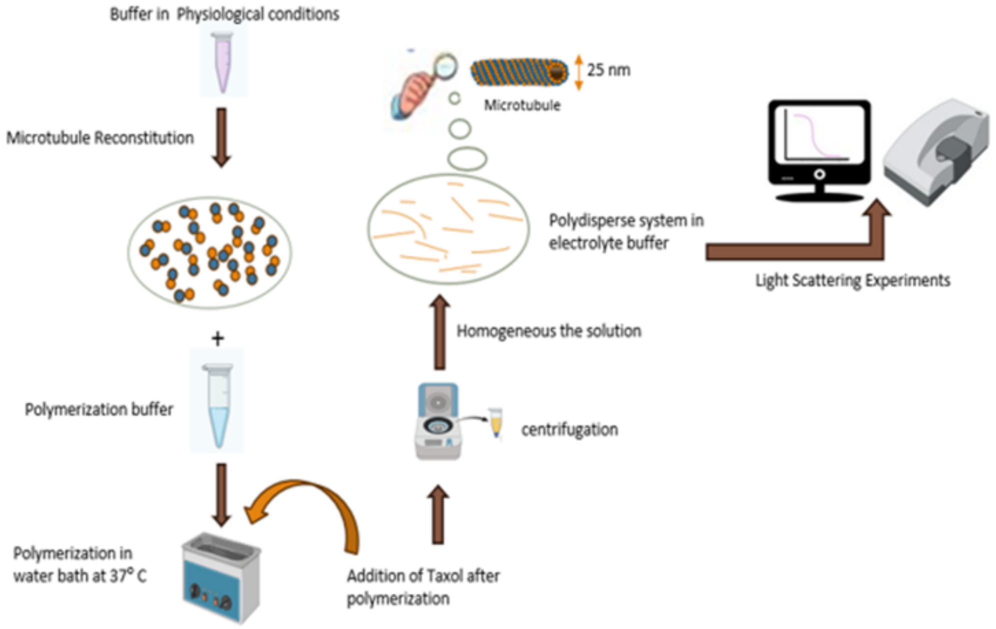

This sample preparation protocol requires 3 days.

All the buffers and solutions are prepared to make enough of four samples of fully developed microtubules in 100 millimolar concentration of potassium chloride (KCl), with a total volume of 1 milliliter of solution. (See Materials sections).

Materials

Materials:

- **Laboratory Equipment/List of Equipment**

1. Analytical Explorer Pro-Scale (Ohaus Industrial Scales, model: PX84)
2. Pipette+ (Sartorius, Andrew Alliance Stand+)
3. Grant SUB Aqua 12 Plus water bath.
4. Smart electronic pipettes: 5–350 µL, 10–1,000 µL, 5–10 mL (Sartorius, Andrew Alliance Stand+)
5. Vortex mixer with standard tube head, 120V (Corning LSE, The Lab Depot, Ref:6775)
6. Orion Star A211 pH meter, accuracy: ± 0.002 (Thermo Scientific, catalog number: X56954)
7. Allegra 64R benchtop centrifuge machine (Beckman Coulter, product number: 367585) with an F1202 Rotor, 30,000 rpm (Beckman Coulter, catalog number: 19U313
8. Zetasizer ULTRA, accuracy MW: ± 10% typical, temperature accuracy: 0.1 °C (Malvern Panalytical, model: ZSU5700)
9. 2 mL glass vial (Agilent, catalog number: 20097845)
10. Polypropylene centrifuge tube, 15 and 50 mL (Corning, catalog number: 430791)
11. Polypropylene microfuge tube, 11 × 38 mm, 1.5 mL (Beckman Coulter, catalog number: 9080511)
12. 12 mm square polystyrene cell cuvettes (Malvern Panalytical, number: DTS0012)
13. 2 mL cryogenic vials (Corning, catalog number: 30721070)
14. Business source stainless steel scissors (Fiskars, catalog number: 01-004250J)
15. Optifit pre-sterilized tips, 10–1,000 µL (Sartorius, catalog number: PR151159)
16. Optifit pre-sterilized tips, 5–350 µL (Sartorius, catalog number: PR149531)
17. 5 3/4” pipets (Fisherbrand, Fisher Scientific, catalog number: 13-678-20A)
18. Universal dip cell kit (Malvern Panalytical, catalog number: ZEN1002)

- **Biological Material**

1. Tubulin Protein (>99% Pure): Porcine Brain; store at 4 °C (Cytoskeleton, catalog number: T240)
2. Guanosine 5’-triphosphate sodium salt (GTP); store at 4 °C (Cytoskeleton, catalog number: BST06)
3. Paclitaxel (Taxol) **Cytoskeleton Inc. Catalog #**TXD01 store at 4 °C

- **Chemicals and Reagents**

1. Dimethyl sulfoxide (DMSO) (Sigma-Aldrich, catalog number: 67-68-5)
2. Water for molecular biology (Millipore, catalog number: H20MB1001)
3. Magnesium chloride, anhydrous, 99% (MgCl2) (Alfa Aesar, catalog number: W07D102)
4. Potassium chloride (KCl) (VWR, BDH Chemicals, catalog number: 18J2256059)
5. Potassium hydroxide (powder) (KOH) (Sigma-Aldrich, catalog number: 1310-58-3)
6. PIPES (piperazine-N, N´-bis (2-Ethane sulfonic acid)) (Molecular biology reagent, catalog number: 194838)
7. EGTA (ethylene glycol-bis (βaminoethyl ether)-N, N, N´, N´-tetra acetic acid) (Sigma -Aldrich, catalog number: E3889-25g)
8. Glycerol, ACS reagent, ≥¬ 99.5% (Sigma-Aldrich, catalog number: 56-81-5)
9. HCl volumetric standard, 0.1 N solution in water (Sigma-Aldrich, catalog number: 7647-01-0)
10. 70% v/v denatured ethanol solution (Fisher Bioreagents, catalog number: 216731)

Buffers:

1. Orion buffers pH 4.01, pH 7.00, and pH 10.01 (Thermo Scientific, catalog numbers: YX1, YW1, YX1)
2. 100 mM PIPES

A	B	C
**Reagent**	**Final concentration**	**Amount**
PIPES	100 mM	302.4 mg
H2O ultra-pure	n/a	9.3-9.5 mL
Total	100 mM	10 mL

3. 10 mM EGTA

A	B	C
**Reagent**	**Final concentration**	**Amount**
EGTA	10 mM	38.04 mg
H2O ultra-pure	n/a	9.6-9.7 mL
Total	10 mM	10 mL

4. 50 mM $MgCl_2$

A	B	C
**Reagent**	**Final concentration**	**Amount**
MgCl2	50 mM	47.61 mg
H2O ultra-pure	n/a	10 mL
Total	50 mM	10 mL

5. 117.47 mM KCl

A	B	C
**Reagent**	**Final concentration**	**Amount**
KCl	117.47 mM	87.52 mg
H2O ultra-pure	n/a	10 mL
Total	117.47 mM	10 mL

6. PEM buffer (pH 7.00)

A	B	C
**Reagent**	**Final concentration**	**Amount**
PIPES	80 mM	4.8 mL
MgCl2	2 mM	240 µL
EGTA	0.5 mM	300 µL
H2O ultra-pure	n/a	660 µL
Total	n/a	6 mL

7. Cushion buffer (pH 7.00)

A	B	C
**Reagent**	**Final concentration**	**Amount**
PIPES	80 mM	3.2 mL
MgCl2	2 mM	160 µL
EGTA	0.5 mM	200 µL
H2O ultra-pure	n/a	440 µL
Glycerol	60 % v/v	6 mL
Total	n/a	10 mL

8. Polymerization buffer (pH 7.00)

A	B	C
**Reagent**	**Final concentration**	**Amount**
PEM	n/a	1000 µL
GTP	n/a	20 µL
PEM with glycerol	n/a	166.6 µL
Total	n/a	1.186 mL

9. PEM-T

A	B	C
**Reagent**	**Final concentration**	**Amount**
PEM	n/a	212.4 µL
Taxol (100 µM)	15 µM	37.6 µL
Total	n/a	250 µL

10. Electrolyte (KCl-T) buffer (pH 7.00)

A	B	C
**Reagent**	**Final concentration**	**Amount**
KCl	100 mM	851.2 µL
Taxol (50 µM)	0.5 µM	10 µL
Taxol (100 µM)	3 µM	30 µL
Taxol (200 µM)	10 µM	50 µL
Total	n/a	941.2 µL

First-day procedure: Stock preparations

4m 30s

1

Note

- To maintain the stock buffers biologically active, they must be remade weekly.
- To prepare [M] 100 millimolar (mM) PIPES with a pH close to 6.8, need to add approximately 13-15 drops of [M] 5 Molarity (M) KOH solution before adding water.
- To prepare [M] 10 millimolar (mM) GTA with pH close to 6.9, need to add approximately 1-2 drops of [M] 5 Molarity (M) KOH solution before adding water.
- All the pipetting and titration are done using the instrument Pipette+ (Sartorius, Andrew Alliace Stand+).

2 **100 mM PIPES:**
Add 302.4 mg of PIPES to a 15 mL polypropylene centrifuge tube.

2.1 Add 13-15 drops of [M] 5 Molarity (M) KOH solution to the tube.

2.2 Vortex the solution for 00:00:35 – 00:00:45 at low to medium speed (4/10) until there is no residue. 45s

2.3 Add 9.3 mL - 9.5 mL of H_2O ultra-pure to the tube.

2.4 Vortex the solution for 00:00:35 – 00:00:45 at low to medium speed (4/10). 45s

3 **10 mM EGTA:**
Add 38.04 mg of EGTA to a 15 mL polypropylene centrifuge tube.

3.1 Add 1-2 drops of [M] 5 Molarity (M) KOH solution to the tube.

3.2 Vortex the solution for 00:00:35 – 00:00:45 at low to medium speed (4/10) until there is no residue. 45s

3.3 Add 9.6 mL - 9.7 mL of H_2O ultra-pure to the tube.

3.4 Vortex the solution for 00:00:35 – 00:00:45 at low to medium speed (4/10). 45s

4 **50 mM $MgCl_2$:**

Add 47.61 mg of $MgCl_2$ to a 15 mL polypropylene centrifuge tube.

4.1 Add 10 mL of H_2O ultra-pure to the tube.

4.2 Vortex the solution for 00:00:35 – 00:00:45 at low to medium speed (4/10). 45s

5 **117.47 mM KCl:**

Add 87.52 mg of KCl to a 50 mL polypropylene centrifuge tube.

5.1 Add 10 mL of H_2O ultra-pure to the tube.

5.2 Vortex the solution for 00:00:35 – 00:00:45 at low to medium speed (4/10). 45s

Note

Store all the stock buffers at 4 °C .

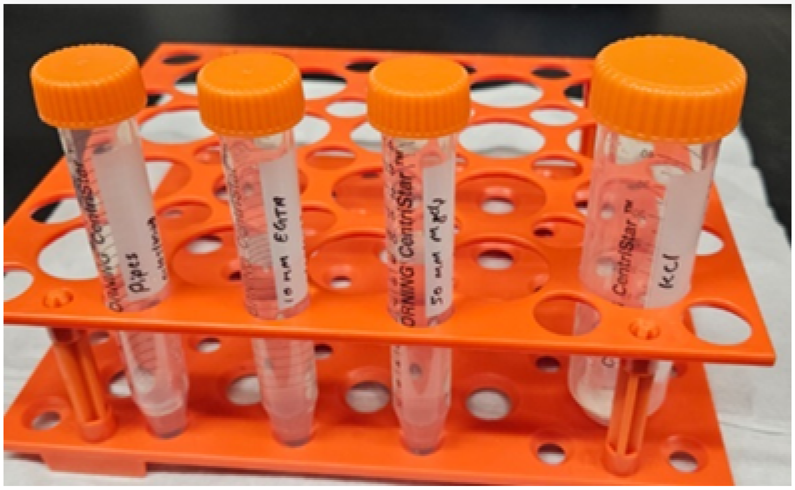

Figure 1: Stock buffers of 100 mM PIPES, 10 mM EGTA, 50 mM $MgCl_2$, and 117.47 mM KCl ready for refrigeration.

Second-day procedure: Tubulin buffers preparations

1m 30s

6 [M] 0 Mass Percent

Note

- Calibrate the pH meter at three-point calibration using Orion buffers pH 4.01 , pH 7.00 , and pH 10.01 before its usage.
- To increase/decrease the pH, add drops of a base ([M] 0.005 Molarity (M) KOH) or acid ([M] 0.1 Mass Percent HCl) solution, respectively. Add these drops carefully and constantly and check the pH, as it can rapidly change.
- Using a 50 mL conical tube was vital since it facilitated measuring and adjusting the pH of the buffers. If the tube is smaller in size, the probe of Orion Star A211 pH meter will not be able to fit; also, there will not be enough solution to cover the tip of the probe leading to inaccurate pH readings. We recommend you consider choosing the size of conical tubes and stock buffer volume based on your pH meter probe size, as microprobes can measure smaller sample volumes effectively.
- When adjusting the pH (PEM, and PEM with glycerol and electrolyte buffers) use glass pipettes to carefully add the base and acid solutions drop by drop into the buffers. Too many drops may significantly increase or decrease the pH, so use caution. It is important to mix well by vortexing the solutions after adding these drops; failing to do so will lead to inaccurate pH readings.

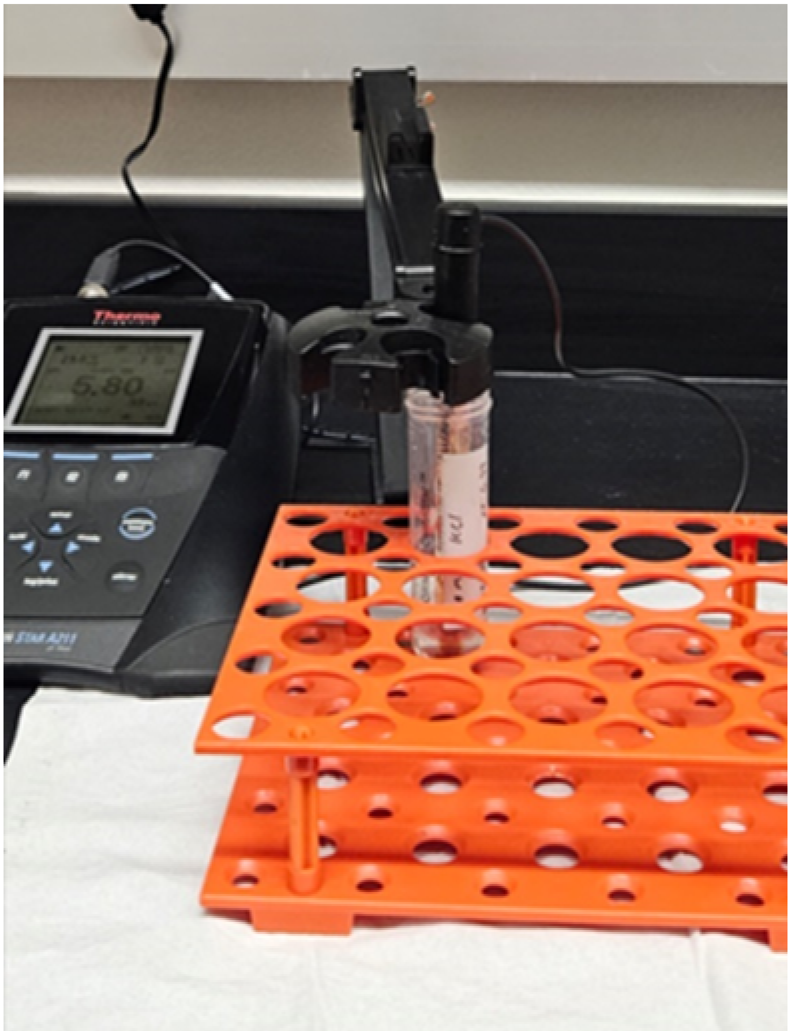

Figure 2: pH meter measuring the pH of [M] 100 millimolar (mM) KCl solution.

7 **PEM or BRB80 or general tubulin buffers (pH 6.91):**
Add 4.8 mL of PIPES to a 50 mL polypropylene centrifuge tube.

7.1 Add 240 µL of $MgCl_2$ to the tube.

7.2 Add 300 µL of EGTA to the tube.

7.3 Add 660 µL of H_2O ultra-pure to the tube.

7.4 Vortex the solution for 00:00:35 – 00:00:45 at low to medium speed (4/10). 45s

8 **PEM with glycerol or cushion buffer or tubulin glycerol buffer (pH 6.97):**
Add 3.2 mL of PIPES to a 50 mL polypropylene centrifuge tube.

8.1 Add 160 µL of $MgCl_2$ to the tube.

8.2 Add 200 µL of EGTA to the tube.

8.3 Add 440 µL of H_2O ultra-pure to the tube.

8.4 Add 6 mL of glycerol to the mixture.

8.5 Vortex the solution for 00:00:35 – 00:00:45 at low to medium speed (4/10). 45s

Note

- Titrate these solutions using the KOH solution with the pH meter to reach the normal pH of pH 7.00 and record it. A pH value of pH 6.90 to pH 7.10 is acceptable for further proceedings.
- Store all these buffers at 4 °C .

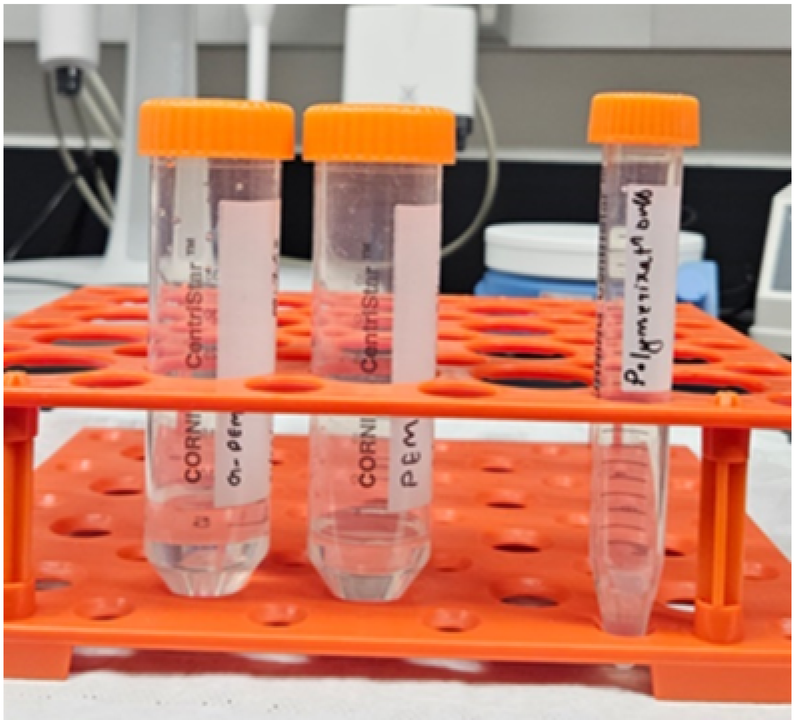

Figure 3: PEM, G-PEM, and polymerization buffer.

Third-day procedure: Polymerization process and suspension buffer

15h 57m 20s

9 **Preparations:**
Before proceeding with the polymerization buffers and the step-by-step procedure, one must ensure to have enough reconstituted GTP, Taxol, and protein. If you already have these, skip to the 'Tubulin polymerization' steps. If not, follow the procedure given below.

10 **a. GTP reconstitution**

> **Note**
>
> - The lyophilized GTP (desiccated to <10% humidity) is stable for six months at 4 °C .
> - The reconstituted GTP is stable if stored at or below -20 °C for six months.
> - All GTP-related steps must be performed with ice in the hood.

10.1 Briefly centrifuge to collect the white powder at the bottom of the storage tube.

10.2 Add 100 µL of ice-cold distilled water for a [M] 100 millimolar (mM) stock solution.

10.3 Aliquot the GTP into experiment-sized amounts as needed.

10.4 Snap-freeze the GTP with liquid Nitrogen.

10.5 Store at or below -20 °C .

11 **b. Taxol reconstitution**

Briefly centrifuge to collect the white powder at the bottom of the storage tube.

11.1 Add 100 µL Dimethyl sulfoxide (DMSO) for a [M] 2 millimolar (mM) stock solution.

11.2 From [M] 2 millimolar (mM) stock solution, pipette 25 µL to a cryotube.

11.3 Add 475 µL of DMSO to the cryotube to make a total of 500 µL of reconstituted Taxol with [M] 100 micromolar (µM) concentration.

11.4 For [M] 200 micromolar (µM) , add 50 µL of Taxol ([M] 2 millimolar (mM)) to 450 µL of DMSO.

11.5 For [M] 100 micromolar (µM) , add 25 µL of Taxol ([M] 2 millimolar (mM)) to 475 µL of DMSO.

11.6 For [M] 50 micromolar (µM) , add 125 µL of Taxol ([M] 200 micromolar (µM)) to 375 µL of DMSO.

11.7 For [M] 10 micromolar (µM) , add 50 µL of Taxol ([M] 100 micromolar (µM)) to 450 µL of DMSO.

11.8 Store at or below -20 °C .

12 **c. Protein reconstitution**

Note

1. Tubulin is a delicate protein; to maintain the integrity and functionality of tubulin, it is crucial to handle it gently and avoid conditions that could cause damage or destabilization mainly because of mechanical stress.
2. The 1 mg tubulin protein powder needs to be extracted to begin the protein reconstitution. If the 1 mg of tubulin is stuck to the bottom of the vial, we suggest vortexing the protein vial at a low to medium speed (3/10) for 00:00:30 – 00:00:45 to smooth the protein and allow easy extraction when the protein is still dry.
3. To avoid repeated thawing cycles, consider specific experimental-sized amounts.
4. Some of the steps considered in this section are recommended by the manufacturer (Cytoskeleton, Inc.). In addition, we considered the addition of mixing steps (12.3 and 12.5), due to the rapid agglomeration/aggregation of the tubulin as the buffer is continuously added. Thus, to achieve an aggregate-free solution we implemented the following: the vortex speed is set at low to medium (4 out of 10) for a short amount of time (for 00:00:30 - 00:00:40). This limits the possibility of a mechanical breakdown of the protein.
5. The aliquots containing the protein and tubulin buffers are stable for six months at -80 °C to preserve their biological activity.

12.1 Transfer the protein powder into a 3 mL glass vial.

12.2 Add 100 µL of ultra-pure water into the glass vial containing the protein powder to reconstitute at 10 mg/mL tubulin density.

12.3 Tubulin proteins may concentrate at the solution's surface or walls of the vial. Vortex the solution for 00:00:30 – 00:00:40 at low to medium speed (4/10) to dissolve the protein powder as much as possible. 40s

12.4 Add 1.5 mL of PEM buffer to the protein solution in the glass vial.

12.5 Vortex once more for 00:00:35 – 00:00:45 at low to medium speed (4/10) to dissolve the aggregates as much as possible. If aggregates are still present, pipette and mix the solution to achieve a homogeneous protein solution. 45s

12.6 Aliquot the solution into experimental-sized amounts according to the number of experiments needed. Transfer the small-size solutions into cryotube vials. We recommend aliquoting the protein solutions into multiples of 400 µL (1×400 µL, 2×400 µL, 3×400 µL, …).

12.7 Once the wanted protein solutions are aliquoted, snap-freeze the cryotube vials with liquid nitrogen and immediately store them at -80 °C .

13 **Tubulin suspension or polymerization buffer**

Note

- Have ice ready in a cooler, as the polymerization buffer must be done on ice.
- Since GTP is toxic to inhale, all steps related to GTP are performed in the fume hood with the sash lowered to ½ of its maximum opening (18 inches).

13.1 Add 1 mL of PEM to a 15 mL polypropylene centrifuge tube.

13.2 Add 20 µL of reconstituted GTP to the tube.

13.3 Add 166.6 µL of PEM with glycerol to the tube.

13.4 Vortex the solution for 00:00:35 – 00:00:45 at low to medium speed (4/10). 45s

13.5 Keep the solution On ice .

14 **Tubulin polymerization** 1h 35m

Note

1. GTP and Taxol were initially stored at -80 °C and -20 °C respectively. When ready to prepare the solutions, thaw both solutions. We aliquoted these into experimental-sized amounts, particularly for one-time use.
2. Following the manufacturer's recommendation, after the 01:30:00 centrifuge process, remove the top 90% (231.2 µL) of the supernatant from each microcentrifuge tube by following step 14.19. The amount of translucent pellets left in the microcentrifuge will be 25.8 µL .
3. Before adding the reconstituted Taxol to the polymerized tubulin in the water bath, we recommend incubating them in the water bath for 00:05:00 to avoid thermal shock to the microtubule filaments.
4. When homogenizing the microtubule pellet in a water bath after centrifugation (Steps 14.20 – 14.22), keep PEM-T, and KCl-T in the water bath to avoid thermal shock to the filaments.

14.1 Extract one of the cryotube vials containing 1×400 µL of protein solution from the -80 °C freezer.

14.2 De-frost the cryotube at Room temperature for 00:05:00 . 5m

14.3 Incubate the vial On ice for 01:00:00 to depolymerize tubulin oligomers that may form during storage. 1h

14.4 Extract 100 µL from the cryotube vial and transfer to a 1.5 mL polypropylene microcentrifuge tube.

14.5 Add 100 µL of polymerization buffer to the microcentrifuge tube containing the 200 µL protein solution to start polymerization.

14.6 Vortex the solution for 00:00:40 – 00:00:50 at low speed (3–4/10). 50s

14.7 Place the tubulin solution samples On ice for 00:10:00 to bind GTP. 10m

14.8 Incubate the protein solution in the Grant SUB Aqua 12 Plus water bath at 37 °C for 00:50:00 . 50m

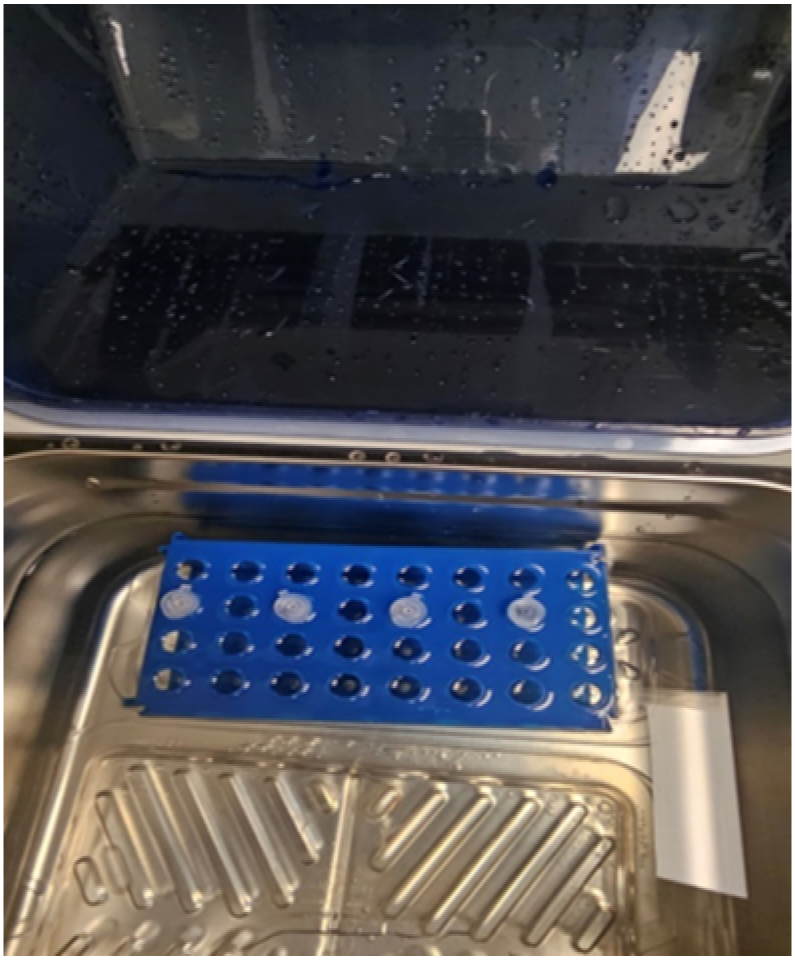

Figure 4: Four tubulin samples immersed in the Grant SUB Aqua 12 Plus water bath at 37 °C for polymerization.

14.9 Set the 5–350 µL Pipette+ in pipette mode at very low speed (1/10) and add 11 µL Taxol from [M] 10 micromolar (µM) stock solution.

14.10 Incubate the samples for 00:10:00 in the water bath. 10m

14.11 Set the 5–350 µL Pipette+ in titration mode at very low speed (1/10) and add 20 µL Taxol from [M] 50 micromolar (µM) stock solution.

14.12 Incubate the samples for 00:10:00 in the water bath. 10m

14.13 Set the 5–350 µL Pipette+ in titration mode at very low speed (1/10) and add 26 µL Taxol from [M] 100 micromolar (µM) stock solution.

14.14 Incubate the samples for the last 00:15:00 in the water bath to reach the stabilization of microtubules with Taxol.

15m

14.15 Turn on the centrifuge 00:10:00 – 00:15:00 before the previous step (14.14) is finalized. Set the centrifuge at 25000 rcf, 25°C, 01:30:00 . The acceleration and deceleration should both be set at a very low (2/10) speed.

1h 45m

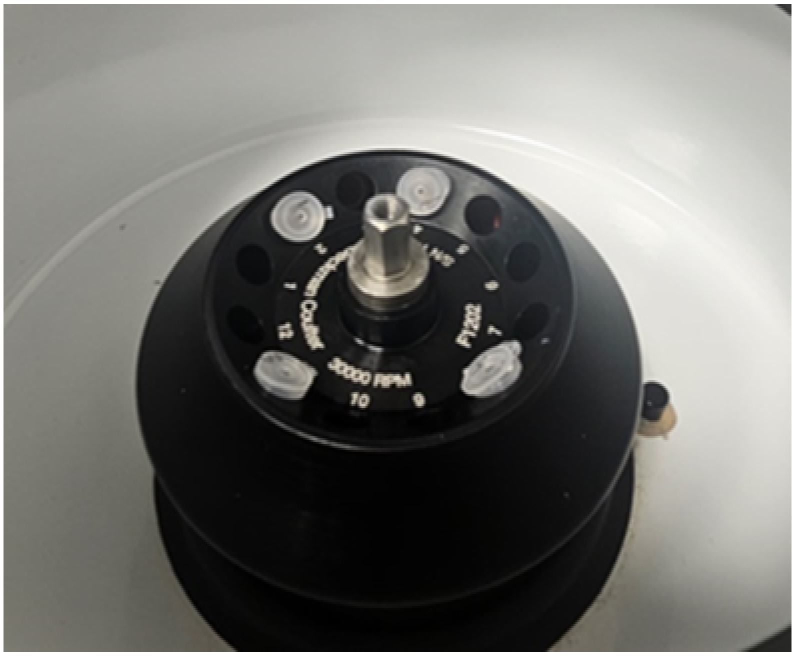

Figure 5: Microtubule samples placed in a centrifuge rotor.

14.16 Centrifuge the protein solutions for 01:30:00 .

1h 30m

14.17 Both PEM-T and KCl-T are prepared during the time of centrifugation.

3m

PEM-T preparation:

1. Add 212.4 µL of PEM buffer to a 1.5 mL polypropylene microcentrifuge tube.
2. Add 37.6 µL of Taxol from [M] 100 micromolar (µM) stock solution.
3. Vortex the solution for 00:00:35 – 00:00:45 at low to medium speed (4/10).
4. Keep the solution at Room temperature .

Electrolyte (KCl-T) buffer preparation:

1. Add 851.2 µL of KCl of [M] 117.47 millimolar (mM) mM of stock solution to a 1.5 mL polypropylene microcentrifuge tube.

2. Add 10 µL of Taxol from [M] 50 micromolar (µM) of stock solution to the KCl solution.
3. Vortex the solution for 00:00:35 – 00:00:45 at low to medium speed (4/10).
4. Add 30 µL of Taxol from [M] 100 micromolar (µM) of stock solution.
5. Vortex the solution for 00:00:35 – 00:00:45 at low to medium speed (4/10).
6. Add 50 µL of Taxol from [M] 200 micromolar (µM) of stock solution.
7. Vortex the solution for 00:00:35 – 00:00:45 at low to medium speed (4/10).
8. h)Keep the solutions at Room temperature .

14.18 After the centrifugation, remove the tubulin protein solution tubes from the centrifuge and place them in a styrofoam vial rack at Room temperature .

14.19 Set the 5–350 µL Pipette+ in titration mode at a very low speed (1/10) and extract the supernatant from the solution in the following manner to avoid any possible stress in the solution that could lead to the breakage of the microtubule filaments: 100 µL, 100 µL, and 31.2 µL.

14.20 Vortex the pellet containing solution for 00:00:20 – 00:00:30 at low speed (3–4/10). 30s

14.21 Incubate the pellet samples for 00:10:00 at 37 °C . 10m

14.22 Add 33 µL of warm PEM-T to pellet samples.

14.23 Keep the pellet samples at Room temperature for 00:10:00 . 10m

14.24 Vortex the solution for 00:00:40 – 00:00:50 at low speed (3–4/10) to make the remaining microtubule pellet (25.8 µL) to reach the homogenous form. 50s

14.25 Using the same Pipette+ specifications as in the previous step (14.24), add 941.2 µL of electrolyte buffer to the microtubule protein pellet (58.8 µL).

14.26 Store the solution at Room temperature and leave it Overnight . 8h

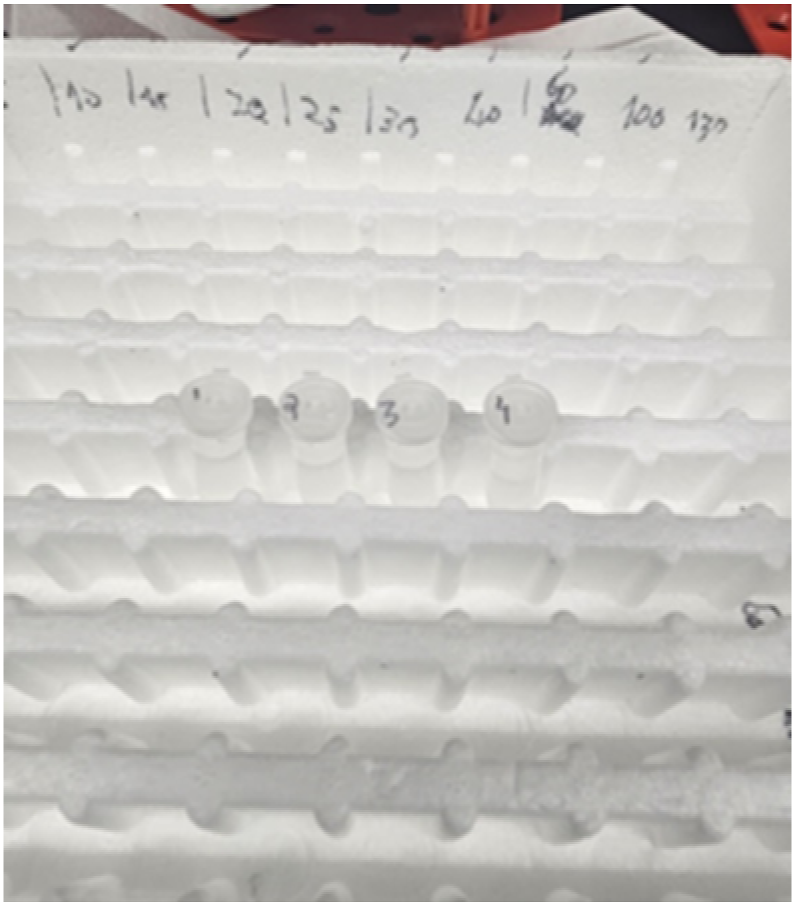

Figure 6: Microtubule samples left overnight to reach the equilibrium with the electrolyte buffer.

14.27 The samples are ready to perform light scattering experiments.

Protocol references

1. E. Alva, A. George, L. Brancaleon and M. Marucho, "Hydrodynamic and Polyelectrolyte Properties of Actin Filaments: Theory and Experiments.," Polymers, vol. 14, no. 12, 6 2022.
2. E. Alva, A. George, L. Brancaleon and M. Marucho, "In vitro Preparation of Homogenous Actin Filaments for Dynamic and Electrophoretic Light Scattering Measurements," Bio-protocol, vol. 12, p. e4553, 2022.
3. J. S. Gethner and F. Gaskin, "Dynamic light scattering from solutions of microtubules.," Biophysical journal, vol. 24, no. 2, pp. 505-15, 11 1978.
4. Y. Jeune-Smith and H. Hess, "Engineering the length distribution of microtubules polymerized in vitro," Soft Matter, vol. 6, no. 8, pp. 1778-1784, 2010.
5. J. C. Lee and S. N. Timasheff, "In vitro reconstitution of calf brain microtubules: effects of solution variables.," Biochemistry, vol. 16, no. 8, pp. 1754-64, 4 1977.
6. M. L. Shelanski, F. Gaskin and C. R. Cantor, "Microtubule assembly in the absence of added nucleotides.," Proceedings of the National Academy of Sciences of the United States of America, vol. 70, no. 3, pp. 765-8, 3 1973.
